# Targeted in vivo epigenome editing of H3K27me3

**DOI:** 10.1186/s13072-019-0263-z

**Published:** 2019-03-13

**Authors:** Hiroto S. Fukushima, Hiroyuki Takeda, Ryohei Nakamura

**Affiliations:** Department of Biological Sciences, Graduate School of Science, The University of Tokyo, Hongo 7-3-1, Bunkyo-ku, Tokyo 113-0033 Japan

**Keywords:** Epigenome editing, H3K27me3, dCas9, Medaka, Transcriptional regulation, Histone modification

## Abstract

**Background:**

Epigenetic modifications have a central role in transcriptional regulation. While several studies using next-generation sequencing have revealed genome-wide associations between epigenetic modifications and transcriptional states, a direct causal relationship at specific genomic loci has not been fully demonstrated, due to a lack of technology for targeted manipulation of epigenetic modifications. Recently, epigenome editing techniques based on the CRISPR-Cas9 system have been reported to directly manipulate specific modifications at precise genomic regions. However, the number of editable modifications as well as studies applying these techniques in vivo is still limited.

**Results:**

Here, we report direct modification of the epigenome in medaka (Japanese killifish, *Oryzias latipes*) embryos. Specifically, we developed a method to ectopically induce the repressive histone modification, H3K27me3 in a locus-specific manner, using a fusion construct of *Oryzias latipes* H3K27 methyltransferase Ezh2 (olEzh2) and dCas9 (dCas9-olEzh2). Co-injection of dCas9-olEzh2 mRNA with single guide RNAs (sgRNAs) into one-cell-stage embryos induced specific H3K27me3 accumulation at the targeted loci and induced downregulation of gene expression.

**Conclusion:**

In this study, we established the in vivo epigenome editing of H3K27me3 using medaka embryos. The locus-specific manipulation of the epigenome in living organisms will lead to a previously inaccessible understanding of the role of epigenetic modifications in development and disease.

**Electronic supplementary material:**

The online version of this article (10.1186/s13072-019-0263-z) contains supplementary material, which is available to authorized users.

## Background

Epigenetic modifications, such as histone modifications and DNA methylation, alter gene transcriptional states, thereby regulating various biological processes (e.g., development, cell differentiation and diseases) [1–3]. Recent studies using next-generation sequencing techniques have revealed genome-wide associations between epigenetic modifications and transcriptional states [4]. However, a lack of technologies for targeted manipulation of histone modifications at individual genomic loci hindered the progress toward demonstrating a causal relationship between specific modifications and their effect on transcriptional regulation.

H3K27me3 is a repressive histone modification and thought to be important for long-term transcriptional repression [1]. In the proposed model of transcriptional repression by H3K27me3, polycomb repressive complex 2 (PRC2) is first recruited to its target sites, and the H3K27 methyltransferase Ezh2 catalyzes H3K27me3. Subsequently, PRC1 binds to H3K27me3 and silences the chromatin [5, 6]. On the other hand, histone acetyltransferase p300 induces H3K27ac, which is associated with open chromatin and transcription factor binding to DNA [7]. Indeed, next-generation sequencing data are generally consistent with these models; H3K27ac is mainly associated with active enhancers, promoters and transcription start sites, while H3K27me3 correlates with repressed or poised promoters and enhancers [4]. The proposed models were based on results from in vitro biochemistry studies, in vivo overexpression, knock-out and knock-down experiments of epigenetic modifying enzymes. However, many of these studies could not exclude the possibility of indirect secondary effects, because such manipulations alter the epigenome globally. Furthermore, recent studies suggested that H3K27me3 could be a consequence of transcriptional repression [8, 9]; PRC1 recruitment can subsequently cause PRC2 protein binding in certain genomic regions [10, 11], and at previously active genes, inhibition of transcription results in the recruitment of PRC2 and accumulation of H3K27me3 [12]. Thus, it is still unclear whether H3K27me3 alone is sufficient to repress gene transcription. H3K27me3 has also been proposed to function as epigenetic memory, which enables the maintenance of a cell-type specific transcriptional state in normal development conditions [2]. However, it is unknown whether histone modifications themselves can be inherited and function as epigenetic memory. Therefore, direct manipulation of H3K27me3 at individual genomic loci is required to fully understand the mechanism of H3K27me3-associated repression.

Targeted manipulation of DNA sequences is one promising approach. Polycomb repressive elements (PREs) were discovered in *Drosophila* [3, 6, 9, 13] and *Arabidopsis thaliana* [14] and are well studied as consensus recruiter sequences that bind PRC2 through interaction with other DNA binding factors. Thus, in such organisms, the deletion or addition of the PRE results in the site-specific reduction or accumulation of H3K27me3 [15, 16]. However, a consensus recruiter sequence like PREs has not been discovered in other organisms such as vertebrates [3]. In addition, in vivo manipulation of DNA sequence requires the establishment of transgenic animals, which remains a time-consuming process. Thus, an alternative technique for in vivo targeted epigenome editing of H3K27me3 is required.

CRISPR-based dCas9 epigenome editing was recently developed as another method for targeted epigenetic manipulation [5]. dCas9 is the nuclease-null deactivated Cas9 which has mutations in the RuvC and HNH domains [17]. Like the CRISPR-Cas9 system, single guide RNA (sgRNA) guides modifying enzymes or domains fused to dCas9 to the targeted genomic locus, which alters the epigenetic state at the site. In principle, this method could be applied to any organism, unlike the deletion of the consensus recruiter sequence. However, the number of editable modifications and reports using the dCas9 system in vivo or in vivo epigenome editing is still limited [18–26].

In this study, we aimed to develop a robust in vivo epigenome manipulation method using medaka (Japanese killifish, *Oryzias latipes*) embryos. We generated a new construct, dCas9-olEzh2 (*Oryzias latipes* Ezh2 fused to dCas9), for manipulating H3K27me3 and demonstrated that dCas9-olEzh2 accumulated H3K27me3 at specific targeted loci and induced gene repression. These in vivo epigenome editing will help the future studies for epigenetic regulation of gene expression and heritability of epigenetic modification at particular genomic loci.

## Results

### dCas9-olEzh2 injection in medaka results in site-specific accumulation of H3K27me3 in vivo

In order to make a new construct for in vivo H3K27me3 manipulation by dCas9 epigenome editing, we first cloned the *Oryzias latipes* H3K27 methyltransferase Ezh2 (olEzh2) sequence and compared it with human, mouse and zebrafish Ezh2 sequences. The alignment revealed that Ezh2 is highly conserved (98%) among the vertebrate species, especially the CXC domain and the SET domain (100%), which are required for H3K27 methyltransferase activity (Additional file 1: Fig. S1).

**Fig. 1:**
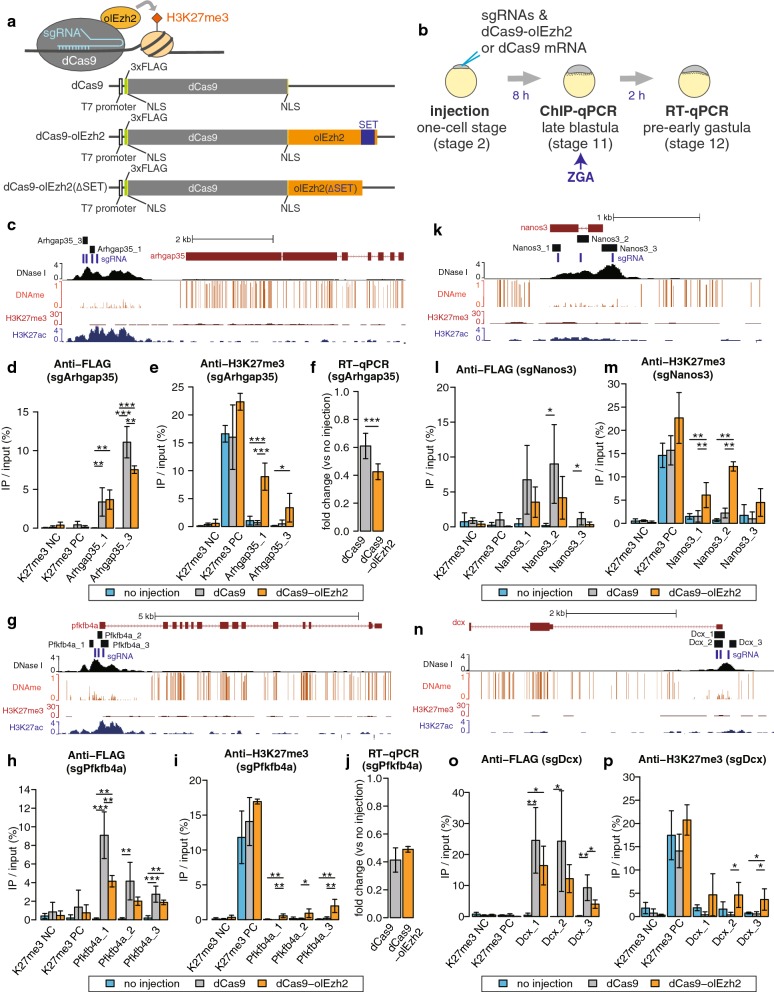
H3K27me3 epigenome editing by dCas9-olEzh2 targeting hypomethylated promoters. **a** Schematic of dCas9, dCas9-olEzh2 and dCas9-olEzh2(∆SET) constructs and H3K27me3 induction caused by dCas9-olEzh2. **b** Schematic view of the dCas9-olEzh2 epigenome editing and injection experiments. sgRNA and mRNA were injected at the one-cell stage (stage 2). ChIP-qPCR was performed using the late blastula embryos (stage 11, 8 h after injection). RT-qPCR was performed using the pre-early gastrula embryos (stage 12, 10 h after injection), because ZGA occurs at the late blastula (stage 11) in medaka. **c**, **g**, **k**, **n** The epigenetic modification patterns around *Arhgap35, Kita*, *Nanos3* and *Dcx*, sgRNAs (blue bars) and ChIP-qPCR product (black bars) positions. H3K27me3 (red) and H3K27ac (blue) ChIP-seq [27], DNase I-seq (black) [28] and DNA methylation [34] enrichment at the blastula stage are shown. **d**, **e**, **h**, **i**, **l**, **m**, **o**, **p** The results of ChIP-qPCR using anti-FLAG antibody (**d**, **h**, **l**, **o**) and anti-H3K27me3 antibody (**e**, **i**, **l**, **m**). H3K27me3 negative region (K27me3 NC) and H3K27me3 positive region (K27me3 PC) were used for ChIP control (described in Additional file 1: Fig. S2). **f**, **j** *Arhgap35* and *Pfkfb4a* mRNA expression fold change. After expression levels were normalized to that of beta-actin, fold changes (sample/no injection) were calculated. Light blue, gray and orange bars in each bar graph represent no injection, sgRNAs/dCas9 injection and sgRNAs/dCas9-olEzh2 injection, respectively. (Tukey–Kramer test and only in Fig. 1f, j Student’s *t* test, *p < 0.1, **p < 0.05, ***p < 0.01, *n* = 3 biological replicates and only in Fig. 1f, j *n* = 6 biological replicates, error bars are s.d., *p* values of each comparison are shown only if the *p* value is under 0.1.)

**Fig. 2:**
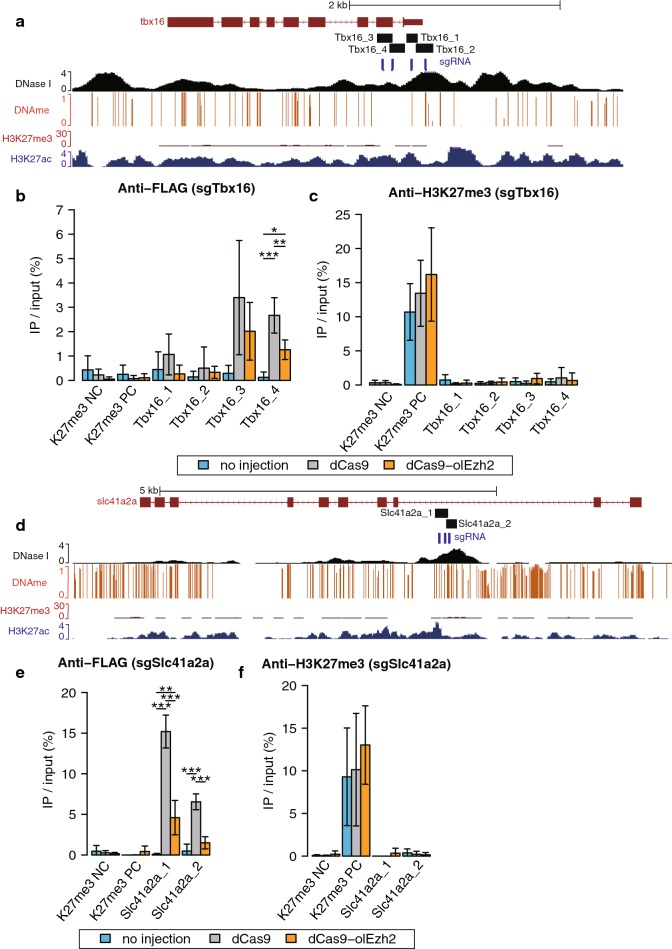
H3K27me3 epigenome editing by dCas9-olEzh2 targeting methylated promoters. **a**, **d** The epigenetic modification patterns around *Tbx16* and *Slc41a2a*, sgRNAs (blue bars) and ChIP-qPCR product (black bars) positions. H3K27me3 (red) and H3K27ac (blue) ChIP-seq [27], DNase I-seq (black) [28] and DNA methylation enrichment [34] at the blastula stage are shown for comparison. **b**, **c**, **e**, **f** The results of ChIP-qPCR using anti-FLAG antibody (**b**, **e**) and anti-H3K27me3 antibody (**c**, **f**). H3K27me3 negative region (K27me3 NC) and H3K27me3 positive region (K27me3 PC) were used for ChIP control (described in Additional file 1: Fig. S2). Light blue, gray and orange bars represent no injection, sgRNAs/dCas9 injection and sgRNAs/dCas9-olEzh2 injection, respectively. (Tukey–Kramer test, *p < 0.1, **p < 0.05, ***p < 0.01, *n* = 3 biological replicates, error bars are s.d., *p* values of each comparison are shown only if the *p* value is under 0.1.)

**Fig. 3:**
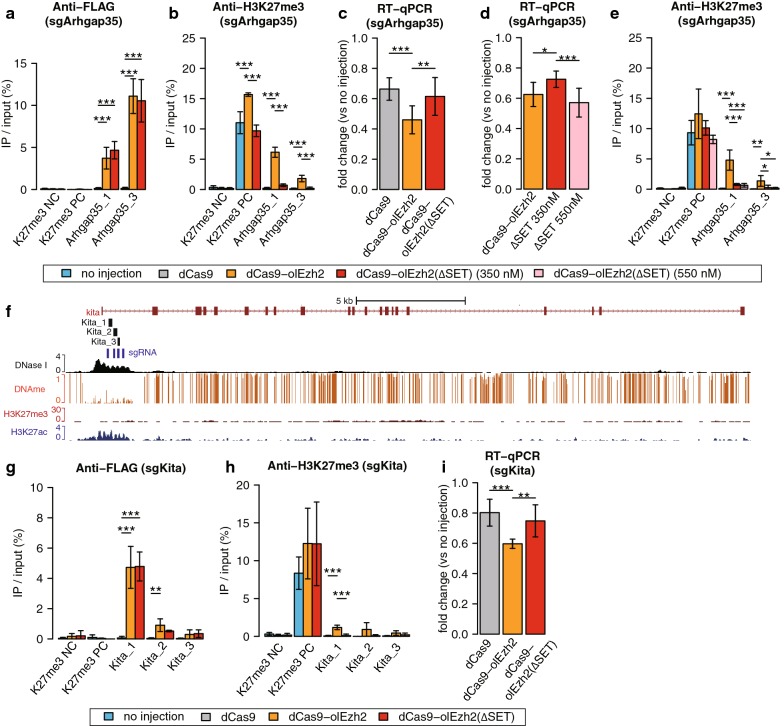
H3K27me3 epigenome editing by dCas9-olEzh2(∆SET) and higher concentration injection. **a**–**c** dCas9-olEzh2(∆SET) analyses targeting *Arhgap35* promoter. ChIP-qPCR using anti-FLAG antibody (**a**), ChIP-qPCR using anti-H3K27me3 antibody (**b**) and *Arhgap35* mRNA expression fold change (**c**). **d**–**e** Arhgap35 mRNA expression fold change (**d**) and ChIP-qPCR using anti-H3K27me3 antibody (**e**) in higher concentration injection. **f** The epigenetic modification patterns around *Kita*, sgRNAs (blue bars) and ChIP-qPCR product (black bars) positions. H3K27me3 (red) and H3K27ac (blue) ChIP-seq [27], DNase I-seq (black) [28] and DNA methylation enrichment [34] at the blastula stage are shown for comparison. **g**–**i** dCas9-olEzh2(∆SET) analyses targeting *Kita* promoter. ChIP-qPCR using anti-FLAG antibody (**g**), ChIP-qPCR using anti-H3K27me3 antibody (**h**) and *Kita* mRNA expression fold change (**i**). In Fig. 4c, e and i, after expression levels were normalized to that of beta-actin, fold changes (sample/no injection) were calculated. Light blue, gray, orange, red and pink bars in each bar graph represent no injection, sgRNAs/dCas9 injection, sgRNAs/dCas9-olEzh2 injection, sgRNAs/dCas9-olEzh2(∆SET)(350 nM) injection and sgRNAs/dCas9-olEzh2(∆SET)(550 nM) injection, respectively. (Tukey–Kramer test, *p < 0.1, **p < 0.05, ***p < 0.01, n = 3 biological replicates and only in **c**, **d** and **i** *n* = 6 biological replicates, error bars are s.d., *p* values of each comparison are shown only if the *p* value is under 0.1.)

To test the ability of olEzh2 to induce H3K27me3 site specifically in vivo, full-length olEzh2 was fused to dCas9 with a FLAG tag at the *N*-terminus (Fig. 1a). To select target genome regions for H3K27me3 manipulation, we investigated our published ChIP-seq data from medaka blastula embryos [27]. We selected promoter regions of 7 genes, *Arhgap35, Pfkfb4a*, *Nanos3*, *Dcx*, *Tbx16, Slc41a2a* and *Kita* as targets, because they showed low H3K27me3 enrichment at the blastula stage (Figs. 1c, g, k, n, 2a, d, 3f). These target promoters do not show any particular characteristics in terms of CpG contents compared to others. sgRNAs were designed to target DNase I hypersensitive sites using DNase I-seq data from medaka blastula [28], because previous genome-wide Cas9 binding studies showed that chromatin inaccessibility prevents sgRNA/Cas9 complex binding [29, 30]. We used a set of sgRNAs targeting a single promoter region because previous studies showed that multiple sgRNAs at each target promoter increased the efficiency of epigenome editing [17, 31, 32].

We injected dCas9 or dCas9-olEzh2 mRNA along with three or four sgRNAs into medaka, the one-cell-stage (stage 2) embryos, and to examine the recruitment of dCas9 or dCas9-olEzh2 and accumulation of H3K27me3 at the target regions, we performed ChIP-qPCR at the late blastula (stage 11), when histone modifications have already been accumulated after epigenetic reprogramming [27, 33] (Fig. 1b). For each target promoter, several primer pairs that overlap with sgRNAs were designed for ChIP-qPCR. The positive and negative controls for ChIP experiments are described in Additional file 1: Fig. S2. The results of ChIP-qPCR using anti-FLAG antibody confirmed that dCas9-olEzh2 was recruited specifically to the target sites (Figs. 1d, h, l, o, 2b, e, 3g). Importantly, at *Arhgap35, Pfkfb4a, Nanos3*, *Dcx* and *Kita* loci, the level of H3K27me3 increased in dCas9-olEzh2 injected embryos, as compared to non-injected and dCas9 injected ones (Figs. 1e, i, m, p, 3h), demonstrating that dCas9-olEzh2 is capable of inducing site-specific H3K27me3 in vivo. On the other hand, at *Tbx16* and *Slc41a2a* loci, there was no significant induction of H3K27me3 (Fig. 2c, f), even though dCas9-olEzh2 was recruited to the target site (Fig. 2b, e). We hypothesized that some factors were preventing the accumulation of H3K27me3 at these two loci. Analysis of published whole-genome bisulfite sequencing data from medaka blastula embryos [34] revealed that *Arhgap35*, *Pfkfb4a, Nanos3*, *Dcx* and *Kita* promoters are hypomethylated (Figs. 1c, g, k, n, 3f), whereas *Tbx16* and *Slc41a2a* promoters are highly methylated (Fig. 2a, d). Antagonism between DNA methylation and H3K27 methylation was previously reported in mouse embryonic stem cells [35] and neural stem cells [36] and also in medaka blastula embryos [27], and therefore, preexisting DNA methylation might have inhibited the induction of H3K27me3 by dCas9-olEzh2 at *Tbx16* and *Slc41a2a* promoters.

Since the antagonism between H3K27me3 and H3K27ac has also been reported [37], we further checked whether the level of H3K27ac was affected by the dCas9-olEzh2-induced H3K27me3 accumulation. However, ChIP-qPCR using anti-H3K27ac antibody at the *Arhgap35* promoter in the sgArhgap35/dCas9-olEzh2 injected embryos showed no significant differences (Additional file 1: Fig. S3), suggesting that the level of H3K27me3 induced by dCas9-olEzh2 was not sufficient for a detectable level of H3K27ac reduction.

### Induced H3K27me3 strengthens site-specific gene repression

Next, we examined whether the induction of H3K27me3 by dCas9-olEzh2 has the function to repress the expression of targeted genes, as H3K27me3 induced by Ezh2 is known as a repressive histone modification [6, 13]. To investigate the repression capacity of dCas9-olEzh2, we chose the zygotically transcribed genes, *Arhgap35*, *Pfkfb4a* and *Kita,* among the five targets that showed H3K27me3 induction. We injected dCas9-olEzh2 mRNA along with sgRNAs targeting the *Arhgap35,* the *Pfkfb4a* or the *Kita* promoter, and performed RT-qPCR at the pre-early gastrula stage (stage 12) (Fig. 1b), which follows the zygotic genome activation (ZGA) at the late blastula stage (stage 11) [38]. As a result, both dCas9- and dCas9-olEzh2-injected embryos showed downregulation of *Arhgap35, Pfkfb4a* or *Kita* compared to non-injected ones (Figs. 1f, j, 3i), and this agrees with a previous report indicating that dCas9 itself can interfere with transcriptional elongation, RNA polymerase binding or transcription factor binding [17]. Importantly, the expression of *Arhgap35* and *Kita* in dCas9-olEzh2-injected embryos was significantly lower than that in dCas9-injected ones (Figs. 1f, 3i), suggesting that H3K27me3 have strengthened the repression. On the other hand, the expression level of *Pfkfb4a* did not show significant difference between dCas9- and dCas9-olEzh2 injected embryos (Fig. 1j). Thus, the effect of H3K27me3 accumulation to gene expression may be different between genes or the levels of H3K27me3 accumulation at *Pfkfb4a* promoter was too low (Fig. 1i).

To validate that the H3K27me3 deposition is causative of transcriptional repression of target genes, we generated a SET domain-deleted mutant dCas9-olEzh2(∆SET) (Fig. 1a). First, we confirmed that this construct had no ability to induce H3K27me3 at target sites (Fig. 3a, b, g, h). Then, we found that the expressions of the two target genes, *Arhgap35* and *Kita*, were significantly lower in dCas9-olEzh2 injected embryos than in dCas9 or dCas9-olEzh2(∆SET)-injected ones (Fig. 3c, i). To further test the possibility that transcriptional interference by dCas9 complex caused the H3K27me3 deposition [8, 9], we increased the molecular concentration of dCas9-olEzh2(∆SET) up to 550 nM and inhibited the gene expression at the same level as dCas9-olEzh2 injection. (Note that all other experiment in this paper used 350 nM concentration.) Under this condition, dCas9-olEzh2(∆SET) (550 nM)-injected embryos showed strong reduction in transcription of the targeted gene (Fig. 3d). However, neither dCas9-olEzh2(∆SET) (350 nM)-injected embryos nor dCas9-olEzh2(∆SET) (550 nM)-injected embryos showed the accumulation of H3K27me3 at the target region (Fig. 3e). Thus, we concluded that the deposition of H3K27me3 was caused by the enzymatic activity of dCas9-olEzh2, but not by transcriptional interference.

### H3K27me3 epigenome editing by dCas9-olEzh2 is highly site-specific

**Fig. 4:**
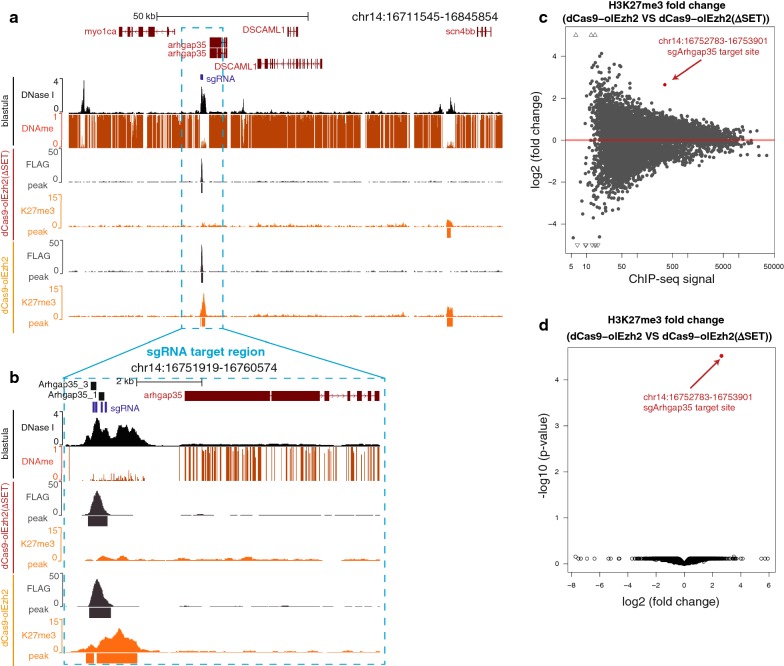
H3K27me3 epigenome editing was highly site specific. **a**, **b** Epigenetic modification patterns, sgRNAs (blue bars) and ChIP-qPCR product (black bars) positions around sgRNA target site. ChIP-seq using anti-FLAG antibody (gray) and anti-H3K27me3 (orange) in dCas9-olEzh2(SET) or dCas9-olEzh2 injected embryos are shown. In addition, DNase I-seq (black) [28] and DNA methylation [34] pattern of blastula stage are shown. **c** MA plot of differential enrichment analysis of ChIP-seq signals of dCas9-olEzh2(SET) injected embryos and dCas9-olEzh2 injected embryos. Each dot shows H3K27me3 peak. The peak with the *p* value under 0.01 is indicated as red dot. The peaks with the fold change greater than 5 or less than − 5 are indicated as triangles. **d** Volcano plot of differential enrichment analysis of ChIP-seq signals of dCas9-olEzh2(SET) injected embryos and dCas9-olEzh2 injected embryos. All H3K27me3 peaks are indicated as dots. Only the peak including targeted genomic region is indicated as red dot

Finally, to globally confirm the specificity of H3K27me3 epigenome editing by dCas9-olEzh2, we performed ChIP-seq of dCas9-olEzh2 mRNA/sgArhgap35 sgRNA-injected or dCas9-olEzh2(∆SET) mRNA/sgArhgap35 sgRNA-injected late blastula (stage 11) embryos using anti-FLAG antibody and anti-H3K27me3 antibody. First, we confirmed that two biological replicates showed consistent distribution of dCas9 binding and H3K27me3 (Additional file 1: Fig. S4a, S4b, S4c, S4d). Thus, in the following analyses, we pooled two replicates. Next, we confirmed the specificity of dCas9-olEzh2(∆SET) and dCas9-olEzh2 recruitment to the target site (Fig. 4a, b, S4a, S4b, S5a, S5b). Finally, we observed that H3K27me3 was only induced at the sgRNA target region in dCas9-olEzh2-injected embryos, while there was no deposition of H3K27me3 in dCas9-olEzh2(∆SET)-injected embryos. Among all H3K27me3 peaks in dCas9-olEzh2(∆SET)- and dCas9-olEzh2-injected embryos, only H3K27me3 enrichment of the sgRNA target region was significantly changed (Fig. 4c, d). These data demonstrate the high specificity of H3K27me3 epigenome editing by dCas9-olEzh2.

## Discussion

Testing so far, the ability of dCas9-olEzh2 to induce H3K27me3 was limited to hypomethylated regions. A previous study using dCas9-PRDM9 (H3K4 methyltransferase PRDM9 fused to dCas9) suggested that dCas9 itself was not able to bind to highly methylated genomic regions [20]. However, our dCas9-olEzh2 successfully bound to methylated target sites. Importantly, we chose the target sites that are DNase I hypersensitive, as previous genome-wide Cas9 binding studies showed that the binding of sgRNA/Cas9 complex depends on chromatin accessibility [29, 30]. Taken together, our results suggest that dCas9-olEzh2 is able to bind to methylated sites if the chromatin is accessible, but the induction of H3K27me3 is prohibited by other inhibitory role of DNA methylation against Ezh2. However, we cannot exclude the possibility that the binding efficiency of sgRNA affected H3K27me3 accumulation to methylated promoters.

Interestingly, the most recent study using human cell lines and mouse Ezh2 fused to the N-terminus of dCas9 (Ezh2-dCas9) reported that H3K27me3 induction at *HER2* promoter did not correlate with transcriptional repression [39]. Also in this study, the two targets (*Arhgap35* and *Kita*) out of the three showed significant downregulation of gene expression, whereas the one (*Pfkfb4a*) of three targets did not. These results suggest that the effect of H3K27me3 on transcription differs among gene loci. Furthermore, the downregulation of target genes (*Arhgap35* and *Kita*), though statistically significant, appeared modest. This suggests that induced H3K27me3 deposition was not sufficient for strong repression under our experimental conditions, or other factors, such as H3K9me or repressor binding, are further required for complete suppression of gene transcription of these genes. In addition, since the deposition of H3K27me3 did not induce the detectable change of H3K27ac level (Additional file 1: Fig. S3), sufficient repression might require de-acetylation.

## Conclusion

In this study, we generated dCas9-olEzh2 for manipulating H3K27me3 and demonstrated that co-injection of three or four sgRNAs and dCas9-olEzh2 mRNA into the one-cell-stage medaka embryos induced accumulation of H3K27me3 at specific targeted loci and significant reduction in gene expression.

Thus far, dCas9-based epigenome editing was reported to site-specifically manipulate H3K27me3 [39], H3K27ac [18], H3K9me3 [19], H3K4me3 [20], H3K79me2 [20] and DNA methylation [21–23] under in vitro conditions. In vivo dCas9-based epigenome editing applications have been used for site-specific deubiquitylation by injection in nuclear transferred *Xenopus* oocyte [25] and targeted manipulation of DNA methylation in mouse oocyte by injection [26] and in mouse brain by in vivo electrophoresis [22, 23]. The present study is the first to site-specifically manipulate H3K27me3 in vivo and extends the applicability of the in vivo dCas9-based epigenome editing. Dysregulation of H3K27me3 has been implicated in diseases such as cancer [40, 41]. Given that Ezh2 is highly conserved among vertebrates including human, our dCas9-olEzh2 system can be a model for in vivo disease treatment in the future.

## Methods

### Medaka strain and developmental stages

Medaka d-rR strain was used for all experiments in this study. Medaka fish were maintained and raised according to standard protocols. Developmental stages were determined based on previously published guidelines [42].

### Cloning and alignment

Total RNA from 2-day post-fertilization medaka embryos was reverse-transcribed to a cDNA mix, using SuperScript^®^ III First-Strand Synthesis SuperMix (Invitrogen, 18080400). Medaka Ezh2 (olEzh2) was amplified from this cDNA mix using cloning primers (described in Additional file 1: Table S1), and PCR products were cloned into the pCR2.1-TOPO vector (pCR2.1-olEzh2). Human, mouse and zebrafish canonical Ezh2 coding DNA sequence (CDS) were obtained from Ensembl (human: ENSP00000419711, mouse: ENSMUSP00000080419, zebrafish: ENSDARP00000023693). These sequences were aligned using T-Coffee [43], and the colored alignment figure was made using the sequence manipulation suite [44].

### sgRNA design

sgRNAs were designed using CCtop CRISPR/Cas9 target online predictor [45] with default parameters except the target site length. We set the target site length to 18. The sgRNA target sequences and locations are described in Additional file 1: Table S1.

### Plasmid construct

Cas9 sequence in pMLM3613 (Addgene, #42251) was modified (D10A and H840A) by PrimeSTAR^®^ Mutagenesis Basal Kit (Takara, R046A) using mut.1 and mut.2 primers. This dCas9 sequence was amplified by primers containing FLAG and NLS sequences; then, it was assembled with XbaI-linearized pCS2+ vector (pCS2+-dCas9) using NEBuilder HiFi DNA Assembly Master Mix (NEB, E2621). From pCR2.1-olEzh2, olEzh2 sequence was amplified and assembled with XhoI-linearized pCS2+-dCas9 vector (pCS2+-dCas9-olEzh2) using NEBuilder. To make pCS2+-dCas9-olEzh2(∆SET) plasmids, we modified olEzh2 sequence in pCS2+-dCas9-olEzh2 by PrimeSTAR^®^ Mutagenesis Basal Kit (Takara, R046A) using mut-olEzh2-ΔSET primers. We constructed the sgRNA vectors from pDR274 (Addgene, #42250) based on the method described in previous paper [46]. All primers used for construction are described in Additional file 1: Table S2.

### In vitro transcription

dCas9, dCas9-olEzh2 and dCas9-olEzh2(∆SET) mRNA were generated using PCR products from pCS2+-dCas9, pCS2+-dCas9-olEzh2, pCS2+-dCas9-olEzh2(∆SET) as templates, respectively, which contains T7 promoters. mRNA was synthesized using HiScribe T7 ARCA mRNA kit (NEB, E2060S). sgRNAs were synthesized using PCR products of sgRNA vectors as templates and HiScribe™ T7 Quick High Yield RNA Synthesis Kit (NEB, E2050S). RNeasy mini kit (Qiagen, 74104) was used to purify RNA. Primers for PCR amplification of the in vitro transcription template are described in Additional file 1: Table S2.

### RNA injection and ChIP-qPCR

For dCas9-olEzh2 injection, either dCas9-olEzh2 mRNA, dCas9-olEzh2(∆SET) or dCas9 mRNA along with three or four sgRNAs (120 ng/μL each) were injected into the one-cell stage (stage 2) embryos. To roughly normalize the number of molecules per injection (350 nM), dCas9-olEzh2, dCas9-olEzh2(∆SET) and dCas9 mRNA were injected at concentration of 750 ng/μL, 710 ng/μL or 500 ng/μL. For higher concentration injection of dCas9-olEzh2(∆SET), dCas9-olEzh2(∆SET) mRNA (1100 ng/µL, 550 nM) and sgRNAs (180 ng/µL) were injected. After 8 h of incubation, the late blastula (stage 11) embryos were transferred into PBS containing 20 mM sodium butyrate, 1 mM PMSF and 1 × cOmplete™ EDTA-free Protease Inhibitor Cocktail (Roche, 11873580001), and cells were gently dissociated using homogenizer (BMBio, C-3452-2) or gentle pipetting (about 150 embryos for dCas9-olEzh2 injection ChIP and about 50 embryos for dCas9-olEzh2(∆SET) injection ChIP). Subsequently, cells were cross-linked by adding formaldehyde (1% volume per volume final) for 8 min at room temperature and then quenched by adding glycine (200 mM final). After washing with PBS containing 20 mM sodium butyrate, 1 mM PMSF and 1 × protease inhibitor cocktail, cross-linked cells were stored in − 80 °C as dry pellet. For dCas9-olEzh2 injection ChIP, all subsequent procedures were performed as previously described [27]. For dCas9-olEzh2(∆SET) injection ChIP, cross-linked cells were sonicated in a microTUBE AFA Fiber Snap-Cap 6 × 16 mm (Covaris, 520045) using Covaris S220 with optimized parameters (Peak Power : 105, Duty Factor : 4.0, cycles per burst: 200, duration: 750 s), and all subsequent procedures were performed as previously described [27]. Anti-FLAG antibody (Sigma, F3165), anti-histone H3K27ac antibody (abcam, ab4729) and anti-histone H3K27me3 antibody (Millipore, 07-449 for sgArhgap35, sgTbx16, sgNanos3 and sgDcx, or Diagenode, c15410069 for sgArhgap35, sgKita, sgPfkfb4a and sgSlc41a2a) were used for each experiment. All primers for ChIP-qPCR are described in Additional file 1: Table S3.

### RT-qPCR

For sgArhgap35, sgKita and sgPfkfb4a RT-qPCR, either dCas9-olEzh2 mRNA (750 ng/μL), dCas9-olEzh2(∆SET) (710 ng/μL) or dCas9 mRNA (500 ng/μL) was injected along with three or four sgRNAs (120 ng/μL each) into the one-cell stage (stage 2) embryos. For higher concentration, injection of dCas9-olEzh2(∆SET), dCas9-olEzh2(∆SET) mRNA (1100 ng/µL, 550 nM) and sgRNAs (180 ng/µL) were injected. After 10 h of incubation, the pre-early gastrula (stage 12) embryos (50 embryos) were homogenized and all subsequent steps were performed as previously described [27]. All primers for RT-qPCR are described in Additional file 1: Table S4.

### ChIP-seq library preparation and sequencing

We generated two biological replicates for ChIP-seq. ChIP was performed following the protocol described above. After ChIP, ChIP-seq libraries were prepared using KAPA Hyper Prep Kit (KAPA Biosystems, KK8504). All ChIP-seq libraries were sequenced using Illumina HiSeq 1500 system.

### ChIP-seq data processing

First, low-quality reads and adapter-derived sequences were trimmed by Trimmomatic [47]. Second, trimmed reads were aligned to medaka genome (MEDAKA1) using BWA [48]. Third, we removed alignments with mapping quality smaller than 20. Finally, MACS2 [49] was used to call peaks (*q* value < 0.01) and to generate signals per million reads tracks.

### ChIP-seq analysis

To test the correlation of the two biological replicates, reads per kilobase per million mapped reads (RPKM) for each 5 kb bin were calculated and Pearson’s correlation coefficient was calculated.

To check the specificity of dCas9-olEzh2 targeting, we plotted fold-enrichment of FLAG ChIP-seq signals by calculating the ratio between the ChIP sample signals and the local control lambda outputted by MACS2 [49].

To investigate the fold change of H3K27me3 enrichment in peaks in dCas9-olEzh2-injected embryos and dCas9-olEzh2(∆SET) embryos, we followed the procedure described in the previous study [19]. We pooled two replicates, called peaks using MACS2 [49], merged H3K27me3 peaks of each condition using bedtools merge [50], calculated the read number overlapping the merged peaks in each replicates using bedtools intersect [50] and compared H3K27me3 enrichment and fold change using DESeq 2 [51].

### Statistics

The experiments shown in Figs. 1f, 3c, d and i had six biological replicates, ChIP-seq experiments had two biological replicates, and all other experiments in this study had three biological replicates. Student’s *t* test was used to compare two groups in Fig. 1f, j. Tukey–Kramer test was used to compare groups in the ChIP-qPCR and RT-qPCR analyses of all other experiments. Data are expressed as mean ± S.D.

## Additional file

**Additional file 1.** Supplementary Figures S1–S5 and Supplementary Tables S1–S4. **Figure S1.** Ezh2 alignment comparing human, mouse, zebrafish and medaka. **Figure S2.** Location and epigenetic modification patterns of ChIP-qPCR negative control (NC) and positive control (PC). **Figure S3.** H3K27ac ChIP-qPCR ofsgArhgap35 injected embryos. **Figure S4.** Comparison between two biological replicates of ChIP-seq. **Figure S5.** Genome-wide distribution of FLAG ChIP-seq signal. **Table S1.** sgRNA targets. **Table S2.** Primers and oligos. **Table S3.** ChIP-qPCR primers. **Table S4.** RT-qPCR primers.

## Abbreviations

olEzh2: oryzias latipes’s Ezh2
sgRNA: single guide RNA
PRC: polycomb repressive complex
PRE: polycomb repressive element
ZGA: zygotic genome activation
olEzh2(∆SET): SET domain deletion mutant of olEzh2
RPKM: reads per kilobase per million mapped reads

## References

[1] D’Urso A, Brickner JH (2014). Mechanisms of epigenetic memory. Trends Genet.

[2] Henikoff S, Greally JM (2016). Epigenetics, cellular memory and gene regulation. Curr Biol.

[3] Schuettengruber B, Bourbon H-M, Di Croce L, Cavalli G (2017). Genome regulation by polycomb and trithorax: 70 years and counting. Cell.

[4] Thakore PI, Black JB, Hilton IB, Gersbach CA (2016). Editing the epigenome: technologies for programmable transcription and epigenetic modulation. Nat Methods.

[5] Kungulovski G, Jeltsch A (2016). Epigenome editing: state of the art, concepts, and perspectives. Trends Genet.

[6] Steffen PA, Ringrose L (2014). What are memories made of? How polycomb and trithorax proteins mediate epigenetic memory. Nat Rev Mol Cell Biol.

[7] Barnes PJ, Adcock IM, Ito K (2005). Histone acetylation and deacetylation: importance in inflammatory lung diseases. Eur Respir J.

[8] Di Croce L, Helin K (2013). Transcriptional regulation by polycomb group proteins. Nat Struct Mol Biol.

[9] Blackledge NP, Rose NR, Klose RJ (2015). Targeting polycomb systems to regulate gene expression: modifications to a complex story. Nat Rev Mol Cell Biol.

[10] Cooper S, Dienstbier M, Hassan R, Schermelleh L, Sharif J, Blackledge NP (2014). Targeting polycomb to pericentric heterochromatin in embryonic stem cells reveals a role for H2AK119u1 in PRC2 recruitment. Cell Rep.

[11] Blackledge NP, Farcas AM, Kondo T, King HW, McGouran JF, Hanssen LLP (2014). Variant PRC1 complex-dependent H2A ubiquitylation drives PRC2 recruitment and polycomb domain formation. Cell.

[12] Riising EM, Comet I, Leblanc B, Wu X, Johansen JV, Helin K (2014). Gene silencing triggers polycomb repressive complex 2 recruitment to CpG Islands genome wide. Mol Cell.

[13] Schuettengruber B, Chourrout D, Vervoort M, Leblanc B, Cavalli G (2007). Genome regulation by polycomb and trithorax proteins. Cell.

[14] Xiao J, Jin R, Yu X, Shen M, Wagner JD, Pai A (2017). Cis and trans determinants of epigenetic silencing by polycomb repressive complex 2 in arabidopsis. Nat Genet.

[15] Laprell F, Finkl K, Müller J (2017). Propagation of polycomb-repressed chromatin requires sequence-specific recruitment to DNA. Science.

[16] Coleman RT, Struhl G (2017). Causal role for inheritance of H3K27me3 in maintaining the OFF state of a drosophila HOX gene. Science.

[17] Qi LS, Larson MH, Gilbert LA, Doudna JA, Weissman JS, Arkin AP (2013). Repurposing CRISPR as an RNA-guided platform for sequence-specific control of gene expression. Cell.

[18] Hilton IB, D’Ippolito AM, Vockley CM, Thakore PI, Crawford GE, Reddy TE (2015). Epigenome editing by a CRISPR-Cas9-based acetyltransferase activates genes from promoters and enhancers. Nat Biotechnol.

[19] Thakore PI, D’Ippolito AM, Song L, Safi A, Shivakumar NK, Kabadi AM (2015). Highly specific epigenome editing by CRISPR-Cas9 repressors for silencing of distal regulatory elements. Nat Methods.

[20] Cano-Rodriguez D, Gjaltema RAF, Jilderda LJ, Jellema P, Dokter-Fokkens J, Ruiters MHJ (2016). Writing of H3K4Me3 overcomes epigenetic silencing in a sustained but context-dependent manner. Nat Commun.

[21] Amabile A, Migliara A, Capasso P, Biffi M, Cittaro D, Naldini L (2016). Inheritable silencing of endogenous genes by hit-and-run targeted epigenetic editing. Cell.

[22] Liu XS, Wu H, Ji X, Stelzer Y, Wu X, Czauderna S (2016). Editing DNA methylation in the mammalian genome. Cell.

[23] Morita S, Noguchi H, Horii T, Nakabayashi K, Kimura M, Okamura K (2016). Targeted DNA demethylation in vivo using dCas9–peptide repeat and scFv–TET1 catalytic domain fusions. Nat Biotechnol.

[24] Lin S, Ewen-Campen B, Ni X, Housden BE, Perrimon N (2015). In vivo transcriptional activation using CRISPR/Cas9 in drosophila. Genetics.

[25] Jullien J, Vodnala M, Pasque V, Oikawa M, Miyamoto K, Allen G (2017). Gene resistance to transcriptional reprogramming following nuclear transfer is directly mediated by multiple chromatin-repressive pathways. Mol Cell.

[26] Yamazaki T, Hatano Y, Handa T, Kato S, Hoida K, Yamamura R (2017). Targeted DNA methylation in pericentromeres with genome editing-based artificial DNA methyltransferase. PLoS ONE.

[27] Nakamura R, Tsukahara T, Qu W, Ichikawa K, Otsuka T, Ogoshi K (2014). Large hypomethylated domains serve as strong repressive machinery for key developmental genes in vertebrates. Development.

[28] Nakamura R, Uno A, Kumagai M, Morishita S, Takeda H (2017). Hypomethylated domain-enriched DNA motifs prepattern the accessible nucleosome organization in teleosts. Epigenetics Chromatin.

[29] Wu X, Scott DA, Kriz AJ, Chiu AC, Hsu PD, Dadon DB (2014). Genome-wide binding of the CRISPR endonuclease Cas9 in mammalian cells. Nat Biotechnol.

[30] Singh R, Kuscu C, Quinlan A, Qi Y, Adli M (2015). Cas9-chromatin binding information enables more accurate CRISPR off-target prediction. Nucleic Acids Res.

[31] Maeder ML, Linder SJ, Cascio VM, Fu Y, Ho QH, Joung JK (2013). CRISPR RNA-guided activation of endogenous human genes. Nat Methods.

[32] Perez-Pinera P, Kocak DD, Vockley CM, Adler AF, Kabadi AM, Polstein LR (2013). RNA-guided gene activation by CRISPR-Cas9-based transcription factors. Nat Methods.

[33] Lindeman LC, Andersen IS, Reiner AH, Li N, Aanes H, Østrup O (2011). Prepatterning of developmental gene expression by modified histones before zygotic genome activation. Dev Cell.

[34] Qu W, Hashimoto SI, Shimada A, Nakatani Y, Ichikawa K, Saito TL (2012). Genome-wide genetic variations are highly correlated with proximal DNA methylation patterns. Genome Res.

[35] Brinkman AB, Gu H, Bartels SJJ, Zhang Y, Matarese F, Simmer F (2012). Sequential ChIP-bisulfite sequencing enables direct genome-scale investigation of chromatin and DNA methylation cross-talk. Genome Res.

[36] Wu H, Coskun V, Tao J, Xie W, Ge W, Yoshikawa K (2010). Dnmt3a-Dependent nonpromoter DNA methylation facilitates transcription of neurogenic genes. Science.

[37] Tie F, Banerjee R, Stratton CA, Prasad-Sinha J, Stepanik V, Zlobin A (2009). CBP-mediated acetylation of histone H3 lysine 27 antagonizes drosophila polycomb silencing. Development.

[38] Aizawa K, Shimada A, Naruse K, Mitani H, Shima A (2003). The medaka midblastula transition as revealed by the expression of the paternal genome. Gene Expr Patterns.

[39] O’Geen H, Ren C, Nicolet CM, Perez AA, Halmai J, Le VM (2017). dCas9-based epigenome editing suggests acquisition of histone methylation is not sufficient for target gene repression. Nucleic Acids Res.

[40] Souroullas GP, Jeck WR, Parker JS, Simon JM, Liu JY, Paulk J (2016). An oncogenic Ezh2 mutation induces tumors through global redistribution of histone 3 lysine 27 trimethylation. Nat Med.

[41] Ezponda T, Licht JD (2014). Molecular pathways: deregulation of histone H3 lysine 27 methylation in cancer—different paths, same destination. Clin Cancer Res.

[42] Iwamatsu T (2004). Stages of normal development in the medaka *Oryzias latipes*. Mech Dev.

[43] Notredame C, Higgins DG, Heringa J (2000). T-coffee: a novel method for fast and accurate multiple sequence alignment. J Thornton J Mol Biol.

[44] Stothard P. The sequence manipulation suite: JavaScript programs for analyzing and formatting protein and DNA sequences. Biotechniques. 2000;28:1102, 1104. http://www.ncbi.nlm.nih.gov/pubmed/10868275. Accessed 27 Nov 2017. 10.2144/00286ir01 10868275

[45] Stemmer M, Thumberger T, Del Sol KM, Wittbrodt J, Mateo JL (2015). CCTop: an intuitive, flexible and reliable CRISPR/Cas9 target prediction tool. PLoS ONE.

[46] Hwang WY, Fu Y, Reyon D, Maeder ML, Tsai SQ, Sander JD (2013). Efficient genome editing in zebrafish using a CRISPR-Cas system. Nat Biotechnol.

[47] Bolger AM, Lohse M, Usadel B (2014). Trimmomatic: a flexible trimmer for Illumina sequence data. Bioinformatics.

[48] Li H, Durbin R (2010). Fast and accurate long-read alignment with Burrows–Wheeler transform. Bioinformatics.

[49] Zhang Y, Liu T, Meyer CA, Eeckhoute J, Johnson DS, Bernstein BE (2008). Model-based analysis of ChIP-Seq (MACS). Genome Biol.

[50] Quinlan AR, Hall IM (2010). BEDTools: a flexible suite of utilities for comparing genomic features. Bioinformatics.

[51] Love MI, Huber W, Anders S (2014). Moderated estimation of fold change and dispersion for RNA-seq data with DESeq2. Genome Biol.

