# Supplementary material for: Targeted in vivo epigenome editing of H3K27me3
Source: Epigenetics Chromatin. 2019 Mar 13;12:17. doi: 10.1186/s13072-019-0263-z (PMC6419334; doi:10.1186/s13072-019-0263-z)
Supplement: Supplementary file 1 — Additional file 1. Supplementary Figures S1–S5 and Supplementary Tables S1–S4. Figure S1. Ezh2 alignment comparing human, mouse, zebrafish and medaka. Figure S2. Location and epigenetic modification patterns of ChIP-qPCR negative control (NC) and positive control (PC). Figure S3. H3K27ac ChIP-qPCR ofsgArhgap35 injected embryos. Figure S4. Comparison between two biological replicates of ChIP-seq. Figure S5. Genome-wide distribution of FLAG ChIP-seq signal. Table S1. sgRNA targets. Table S2. Primers and oligos. Table S3. ChIP-qPCR primers. Table S4. RT-qPCR primers. [file 13072_2019_263_MOESM1_ESM.pdf]

Additional File

Targeted *in vivo* epigenome editing of H3K27me3

Hiroto S. Fukushima[1], Hiroyuki Takeda[1,*] and Ryohei Nakamura[1,*]

[1]Department of Biological Sciences, Graduate School of Science, The University of Tokyo, Hongo 7-3-1, Bunkyo-ku, Tokyo, 113-0033, Japan
*Correspondence to H.T. and R.N.

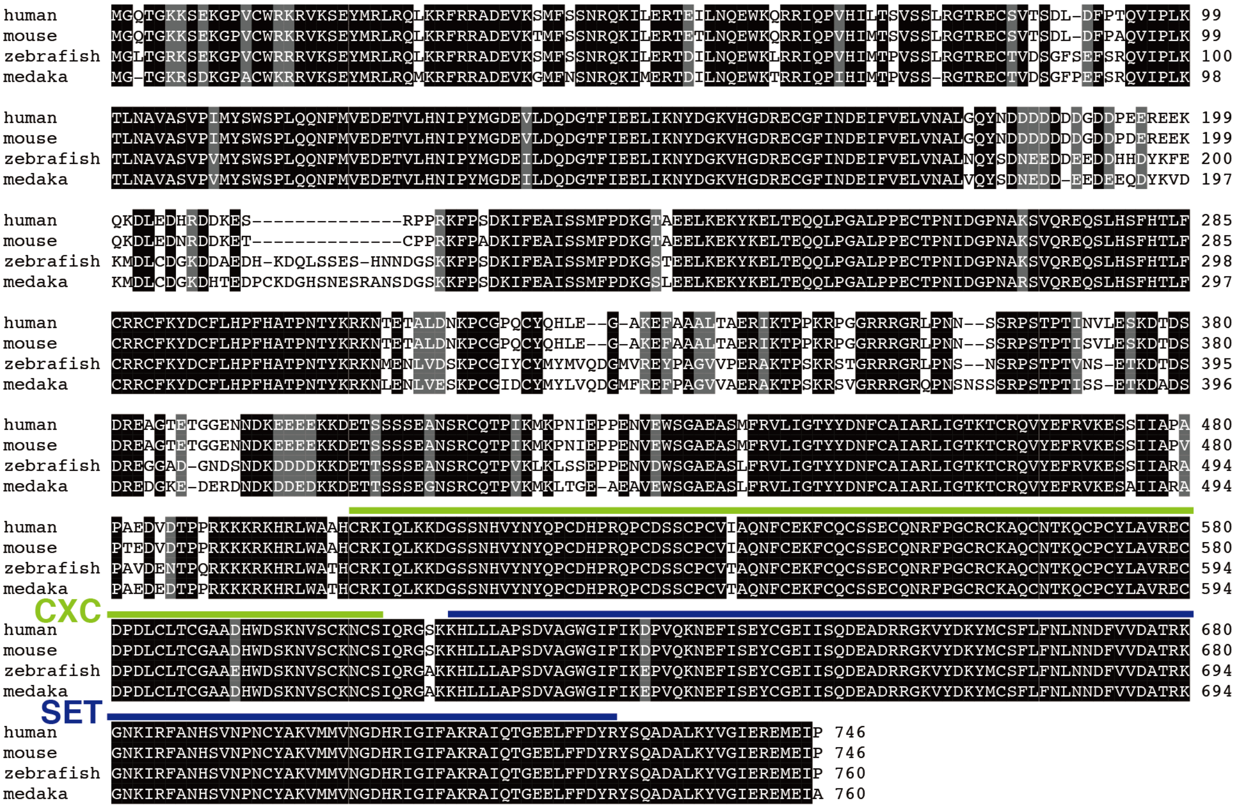

Figure S1. Ezh2 alignment comparing human, mouse, zebrafish and medaka.

Alignment of Ezh2 protein sequences from four species. The green and blue bars indicate the CXC and the SET domains, respectively.

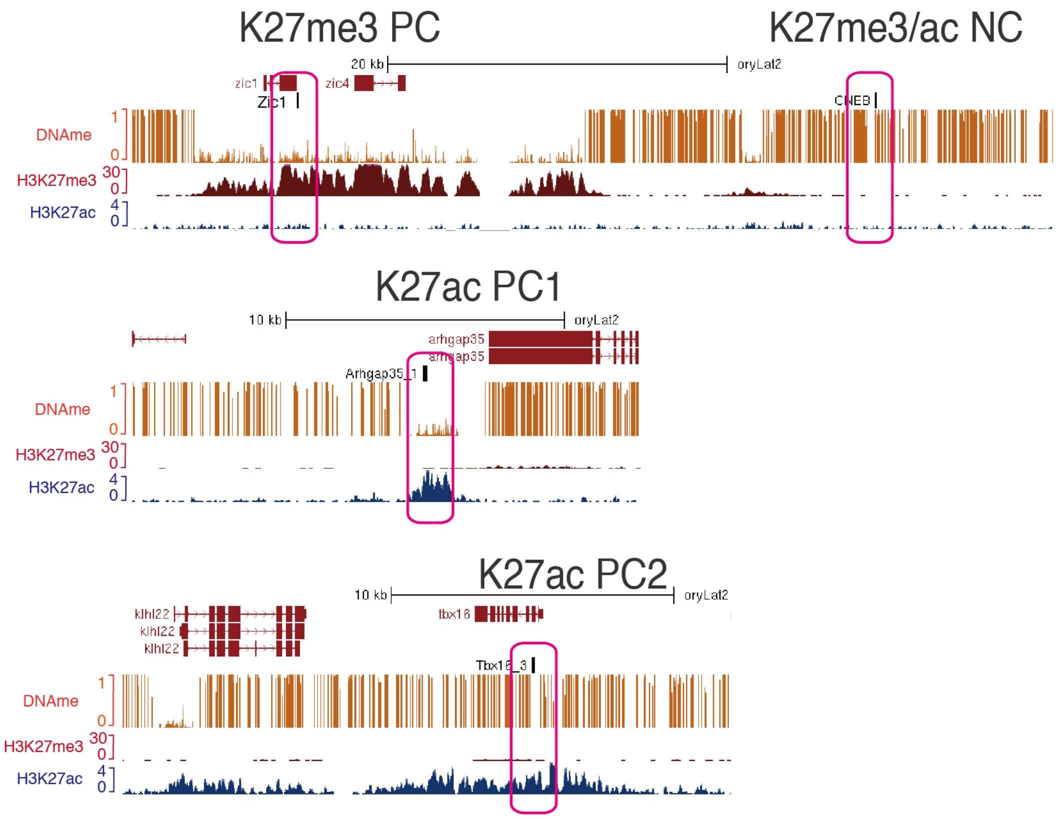

Figure S2. Location and epigenetic modification patterns of ChIP-qPCR negative control (NC) and positive control (PC).

H3K27me3 (red), H3K27ac (blue) enrichment (ChIP-seq)[27] and DNA methylation[34] at the blastula stage are shown. Black bars below the genes surrounded by magenta circles are the ChIP-qPCR product positions of each NC and PC.

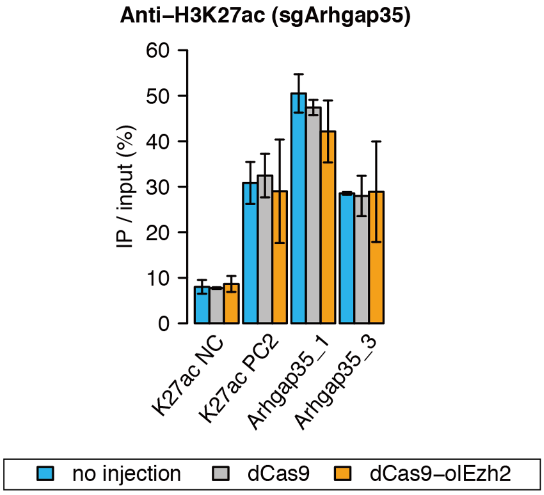

Figure S3. H3K27ac ChIP-qPCR of sgArhgap35 injected embryos.

H3K27ac negative region (K27ac NC) and H3K27ac positive region (K27ac PC2) were used as controls for ChIP (described in Fig. S2). Light blue, gray and orange bars represent no injection, sgRNAs / dCas9 injection and sgRNAs / dCas9-olEzh2 injection, respectively.

Figure S4

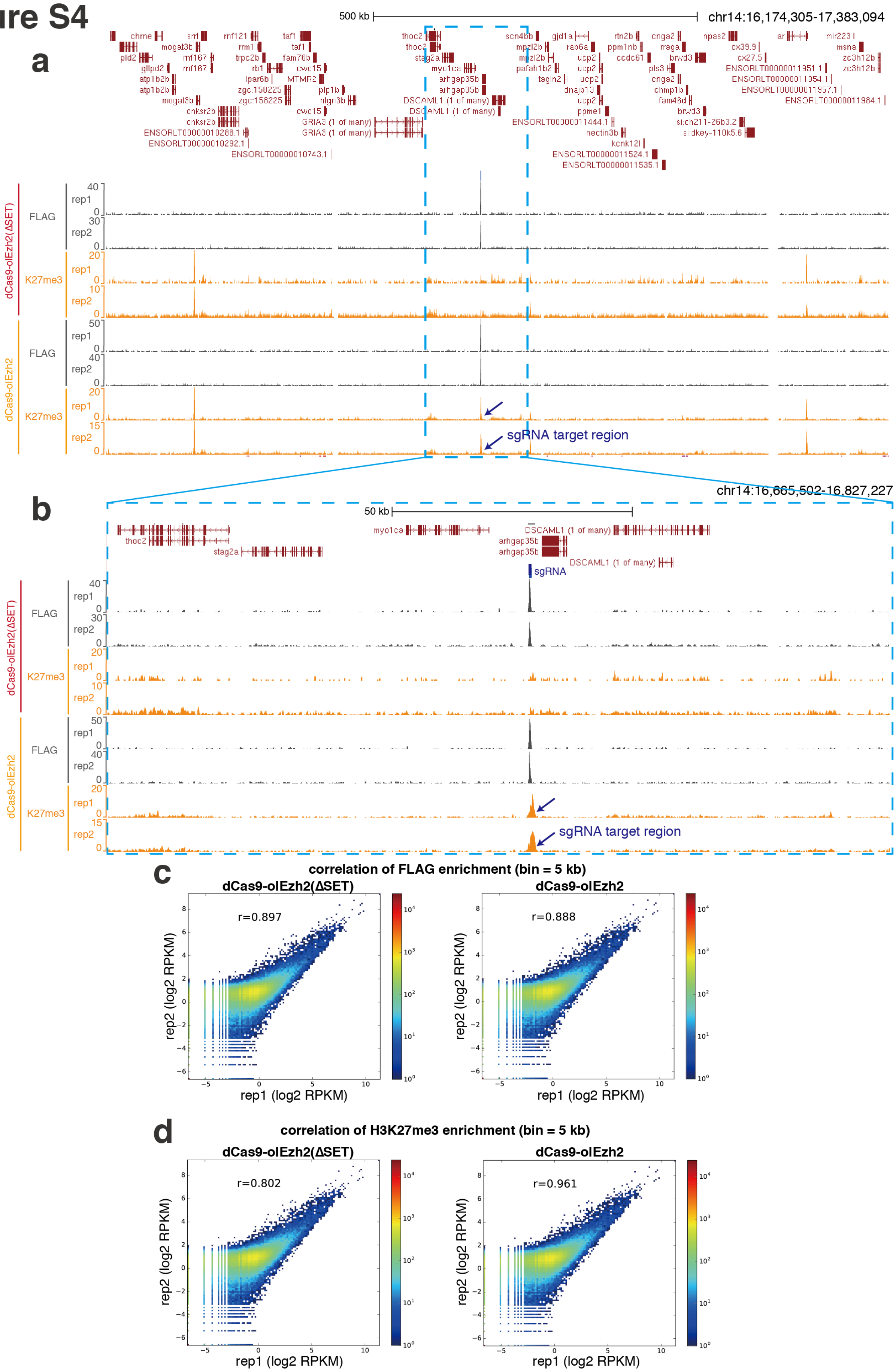
a
500 kb
chr14:16,174,305-17,383,094
dCas9-olEzh2(ΔSET)
dCas9-olEzh2
FLAG
K27me3
rep1
rep2
sgRNA target region
b
chr14:16,665,502-16,827,227
50 kb
sgRNA
c
correlation of FLAG enrichment (bin = 5 kb)
dCas9-olEzh2(ΔSET)
dCas9-olEzh2
r=0.897
r=0.888
rep2 (log2 RPKM)
rep1 (log2 RPKM)
d
correlation of H3K27me3 enrichment (bin = 5 kb)
r=0.802
r=0.961

Figure S4. Comparison between two biological replicates of ChIP-seq.

(a, b) Genome browser view of ChIP-seq for each biological replicate. Induced H3K27me3 accumulation at the target locus is indicated by arrows. **(c, d)** Correlation of two ChIP-seq replicates. Log2(RPKM) of each bins (5kb) and Pearson's correlation coefficient are shown.

Figure S5

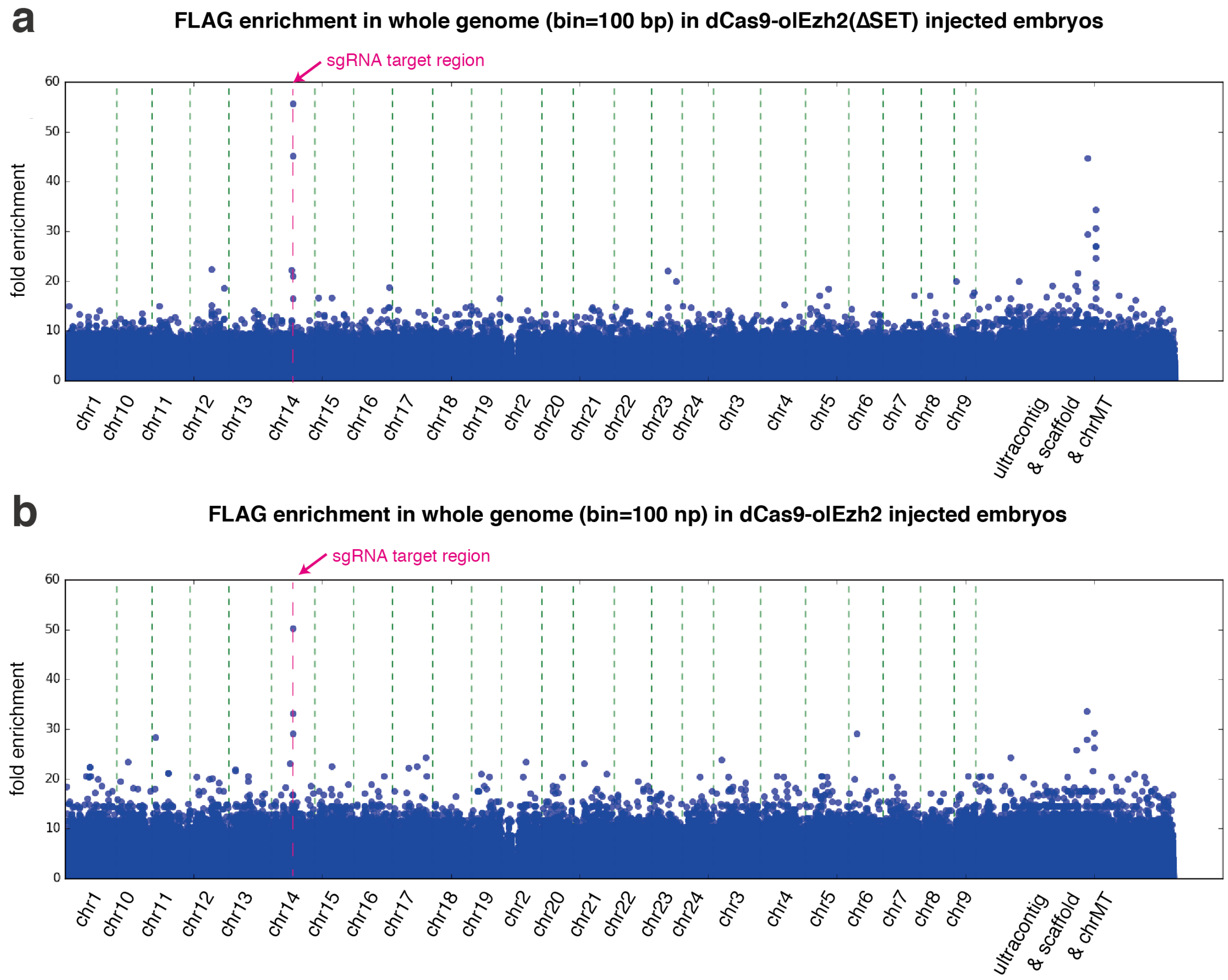

Figure S5. Genome-wide distribution of FLAG ChIP-seq signal.

(a, b) Fold enrichment of FLAG ChIP-seq signal for (a) dCas9-olEzh2(ΔSET) injected embryos and (b) dCas9-olEzh2 injected embryos. The position of sgRNA target site is indicated by magenta line.

Table S1. sgRNA targets

name	sgRNA target (18bp) + NGG	location
**sgArhgap35_1**	ACCACACGATAGCGCCTCTGG	chr14:16752902-16752922
**sgArhgap35_2**	TGCCGACATAATAACGCTGGG	chr14:16753019-16753039
**sgArhgap35_3**	ATGTGGAATCGATTACTTTGG	chr14:16752685-16752705
**sgArhgap35_4**	TTGTGTGAGGGCGCCGCTGGG	chr14:16752757-16752777
**sgPfkfb4a_1**	CGCGCGAGCGCTACCGACTGG	chr5:25992580-25992600
**sgPfkfb4a_2**	TACACAGTCACCGACACGGGG	chr5:25992672-25992692
**sgPfkfb4a_3**	GGGAAGCCCGTTTTTATACGG	chr5:25992789-25992809
**sgTbx16_1**	ACTCAATGTGTAATCCGTGGG	chr9:20564664-20564684
**sgTbx16_2**	GGTGGACGACGACTTTTAAGG	chr9:20564800-20564820
**sgTbx16_3**	CTGGAGGATCCCAACCTCTGG	chr9:20564401-20564421
**sgTbx16_4**	GAGGAGGGCCACTGAAGTTGG	chr9:20564478-20564498
**sgSlc41a2a_1**	GAGTCGTTGCAATCGTCTGGG	chr6:26429286-26429306
**sgSlc41a2a_2**	ACAGATTTAGTGTAGTCGAGG	chr6:26429140-26429160
**sgSlc41a2a_3**	AGCTCGAATCCCTTCTAGTGG	chr6:26429232-26429252
**sgNanos3_1**	GGGCACACGTACTGCCGCAGG	chr1:31476661-31476681
**sgNanos3_2**	CTTCGTGCACCCCCGCTCCGG	chr1:31476915-31476935
**sgNanos3_3**	GCGCGTGCACAGCGCGTGCGG	chr1:31477286-31477306
**sgDcx_1**	CACCTGCGCGGCTGCGTGCGG	chr14:14993085-14993105
**sgDcx_2**	CGGTGCGGTGCGGGGGAAGGG	chr14:14993121-14993141
**sgDcx_3**	GACTCGCACCTCCGCGGTGGG	chr14:14993297-14993317
**sgKita_1**	ACTCCGGGCTCTCTTAACCGG	chr4:1430456-1430476
**sgKita_2**	ACATAACCGTCACTATCATGG	chr4:1430718-1430738
**sgKita_3**	TCAGGTACATAATATACGAGG	chr4:1430924-1430944
**sgKita_4**	GTCATGAAAAGCCACTTCAGG	chr4:1431112-1431132

Table S2. Primers and oligos

name	purpose	Primer Sequence (5'->3')
**olEzh2 F**	cloning olEzh2	TGGCTGCAGGCTGATCAT
**olEzh2 R**	cloning olEzh2	TCAGGCGATCTCCATCTC
**mut.1_F**	dCas9 mutagenesis (D10A)	GGCTTAGCTATCGGCACAAATAGCGT
**mut.1_R**	dCas9 mutagenesis (D10A)	GCCGATAGCTAAGCCTATTGAGTATT
**mut.2_F**	dCas9 mutagenesis (H840A)	TGTCGATGCCATTGTTCCACAAAGTTT
**mut.2_R**	dCas9 mutagenesis (H840A)	ACAATGGCATCGACATCATAATCACT
**NEBuilder dCas9 F**	make pCS2+-dCas9	ATACGACTCACTATAGTTGAGAGCCGCCACCATGGAC
**NEBuilder dCas9 R**	make pCS2+-dCas9	AGGCCTCTCGAGCCTAAACTCAATGGTGATGGTGATGAT GACC
**NEBuilder olezh2 F**	make pCS2+-dCas9-olEzh2	CCCAAGAAGAAGAGGAAAGTCCGCGCCGGGACGGGGA AACGCTCAG
**NEBuilder olezh2 R**	make pCS2+-dCas9-olEzh2	TCGAATTCAAGGCCTCTCAGGCGATCTCCATCTCGC
**mut-olEzh2-ΔSET F**	make Ezh2(ΔSET)	AGCCAAGTACAGCCAGGCGGACGCC
**mut-olEzh2-ΔSET R**	make Ezh2(ΔSET)	TGGCTGTACTTGGCTCCTCTCTGGAT
**IVT dCas9-olezh2 F**	IVT template (dCas9 or dCas9-olEzh2)	GGATCTACGTAATACGACTCACTA
**IVT dCas9-olezh2 R**	IVT template (dCas9 or dCas9-olEzh2)	GCTACTTGTTCTTTTTGCAGG
**IVT sgRNA F**	IVT template (sgRNA)	AAAAGCACCGACTCGGTG
**IVT sgRNA R**	IVT template (sgRNA)	GGTCAGGTATGATTTAAATGGTCAGT
**sgArhgap35_1s**	sgRNA oligo	TAGGACCACACGATAGCGCCTC
**sgArhgap35_1a**	sgRNA oligo	AAACGAGGCGCTATCGTGTGGT
**sgArhgap35_2s**	sgRNA oligo	TAGGTGCCGACATAATAACGCT
**sgArhgap35_2a**	sgRNA oligo	AAACAGCGTTATTATGTCGGCA
**sgArhgap35_3s**	sgRNA oligo	TAGGATGTGGAATCGATTACTT
**sgArhgap35_3a**	sgRNA oligo	AAACAAGTAATCGATTCCACAT
**sgArhgap35_4s**	sgRNA oligo	TAGGTTGTGTGAGGGCGCCGCT
**sgArhgap35_4a**	sgRNA oligo	AAACAGCGGCGCCCTCACACAA

**sgPfkfb4a_1s**	sgRNA oligo	TAGGCGCGCGAGCGCTACCGAC
**sgPfkfb4a_1a**	sgRNA oligo	AAACGTCGGTAGCGCTCGCGCG
**sgPfkfb4a_2s**	sgRNA oligo	TAGGTACACAGTCACCGACACG
**sgPfkfb4a_2a**	sgRNA oligo	AAACCGTGTCGGTGACTGTGTA
**sgPfkfb4a_3s**	sgRNA oligo	TAGGGGGAAGCCCGTTTTTATA
**sgPfkfb4a_3a**	sgRNA oligo	AAACTATAAAAACGGGCTTCCC
**sgTbx16_1s**	sgRNA oligo	TAGGACTCAATGTGTAATCCGT
**sgTbx16_1a**	sgRNA oligo	AAACACGGATTACACATTGAGT
**sgTbx16_2s**	sgRNA oligo	TAGGGGTGGACGACGACTTTTA
**sgTbx16_2a**	sgRNA oligo	AAACTAAAAGTCGTCGTCCACC
**sgTbx16_3s**	sgRNA oligo	TAGGCTGGAGGATCCCAACCTC
**sgTbx16_3a**	sgRNA oligo	AAACGAGGTTGGGATCCTCCAG
**sgTbx16_4s**	sgRNA oligo	TAGGGAGGAGGGCCACTGAAGT
**sgTbx16_4a**	sgRNA oligo	AAACACTTCAGTGGCCCTCCTC
**sgSlc41a2a_1s**	sgRNA oligo	TAGGGAGTCGTTGCAATCGTCT
**sgSlc41a2a_1a**	sgRNA oligo	AAACAGACGATTGCAACGACTC
**sgSlc41a2a_2s**	sgRNA oligo	TAGGACAGATTTAGTGTAGTCG
**sgSlc41a2a_2a**	sgRNA oligo	AAACCGACTACACTAAATCTGT
**sgSlc41a2a_3s**	sgRNA oligo	TAGGAGCTCGAATCCCTTCTAG
**sgSlc41a2a_3a**	sgRNA oligo	AAACCTAGAAGGGATTCGAGCT
**sgNanos3_1s**	sgRNA oligo	TAGGGGGCACACGTACTGCCGC
**sgNanos3_1a**	sgRNA oligo	AAACGCGGCAGTACGTGTGCCC
**sgNanos3_2s**	sgRNA oligo	TAGGCTTCGTGCACCCCCGCTC
**sgNanos3_2a**	sgRNA oligo	AAACGAGCGGGGGTGCACGAAG
**sgNanos3_3s**	sgRNA oligo	TAGGGCGCGTGCACAGCGCGTG
**sgNanos3_3a**	sgRNA oligo	AAACCACGCGCTGTGCACGCGC
**sgDcx_1s**	sgRNA oligo	TAGGACCTGCGCGGCTGCGTGC
**sgDcx_1a**	sgRNA oligo	AAACGCACGCAGCCGCGCAGGT
**sgDcx_2s**	sgRNA oligo	TAGGCGGTGCGGTGCGGGGGAA
**sgDcx_2a**	sgRNA oligo	AAACTTCCCCCGCACCGCACCG
**sgDcx_3s**	sgRNA oligo	TAGGGACTCGCACCTCCGCGGT
**sgDcx_3a**	sgRNA oligo	AAACACCGCGGAGGTGCGAGTC
**sgKita_1s**	sgRNA oligo	TAGGACTCCGGGCTCTCTTAAC
**sgKita_1a**	sgRNA oligo	AAACGTTAAGAGAGCCCGGAGT

**sgKita_2s**	sgRNA oligo	TAGGACATAACCGTCACTATCA
**sgKita_2a**	sgRNA oligo	AAACTGATAGTGACGGTTATGT
**sgKita_3s**	sgRNA oligo	TAGGTCAGGTACATAATATACG
**sgKita_3a**	sgRNA oligo	AAACCGTATATTATGTACCTGA
**sgKita_4s**	sgRNA oligo	TAGGGTCATGAAAAGCCACTTC
**sgKita_4a**	sgRNA oligo	AAACGAAGTGGCTTTTCATGAC

Table S3. ChIP-qPCR primers

name	Forward Primer Sequence (5'->3')	Reverse Primer Sequence (5'->3')	location
**Arhgap35_1**	AGAGGAGATCTCGGTCCAGG	CGTGCCGTTTCCTTCCAAAT	chr14:16752834-16752975
**Arhgap35_3**	ACAAGCTCTCGTGGATGTGG	ACACAAGCAACGGAGAGAGG	chr14:16752671-16752803
**Pfkfb4a_1**	GTTGATGGGCCTGTCCCAAA	GATGTTTCCCGTGATGCTGC	chr5:25992515-25992672
**Pfkfb4a_2**	GCAGCATCACGGGAAACATC	GGGTGAGTTCGCGATGAGTA	chr5:25992653-25992762
**Pfkfb4a_3**	GCCTCTACTCATCGCGAACT	CCAGCTACACAAGACAAAGGC	chr5:25992738-25992922
**Tbx16_1**	CAGCAGCTCTGGCTGAGAAA	CTTTCACACTTGAGGCAGGC	chr9:20564630-20564731
**Tbx16_2**	GCCTGCCTCAAGTGTGAAAG	GGCTCATCACCGTGTCACTT	chr9:20564712-20564886
**Tbx16_3**	ACCTGCCTGGCTTTGTGATA	CAACTTCAGTGGCCCTCCTC	chr9:20564349-20564497
**Tbx16_4**	CAGTGATGGCAGTGGGAGG	TGCAGTCCATCAGAGGTGAG	chr9:20564463-20564615
**Slc41a2a_1**	CCTGCTGGTCGCCACATTTA	CGACTCTGAAGGCGTCAACA	chr6:26429104-26429291
**Slc41a2a_2**	GGTGTTGACGCCTTCAGAGT	CTACGCCACCACCTATCTCC	chr6:26429270-26429412
**Nanos3_1**	AGTTCTGTCCACCTTCGGAC	TGTGTTGTGTCCCTACCTGC	chr1:31476597-31476696
**Nanos3_2**	CAAAGCTACCTGGGAGATGGG	GGGCCATCGTTCAAGCAAAT	chr1:31476881-31477020
**Nanos3_3**	GACATGCTTAGCGCACCTCT	CGTCACGCTTCACCTGTTCA	chr1:31477165-31477345
**Dcx_1**	TAGGAGAATAGGAGTCATGTGGTTT	GAAGCAGACAAAGGCAGAGC	chr14:14993043-14993224
**Dcx_2**	AGGAGAATAGGAGTCATGTGGTTT	CGCTATCCTTCCTGCTGCAT	chr14:14993044-14993189
**Dcx_3**	GGAGGTGCGAGTCTGCG	CAGCCACCACAGCAATTCAT	chr14:14993305-14993422
**Zic1**	CATCAGATGAGCGTTGTAGG	CTGAGACGACTGAGAGCAG	chr20:16773372-16773539
**CNEB**	ACGCTGCATGCATCAAACAAGGC	TGTCACACAACCCGGGCACAC	chr20:16807468-16807603
**Kita_1**	CCCGGAGTAACGAAACCCAA	ATTCTGACCTGTCGGCTTCC	chr4:1430469-1430657
**Kita_2**	AGCTCCTGCTGTCCCTACAT	CATGAATGACCTCTGCGGGT	chr4:1430702-1430899
**Kita_3**	CCGCAGAGGTCATTCATGGT	ACGTCCATGTTTCCTGACTCC	chr4:1430882-1431010

Table S4. RT-qPCR primers

name	Forward Primer Sequence (5'->3')	Reverse Primer Sequence (5'->3')
**Arhgap35**	TGAAGAGCCTCAGACGAACAG	TACGCGGTACAAACCCTCTG
**Pfkfb4a**	CATCGCGAACTCACCCAGAA	CCGATCCAGTTCAGGTAGCG
**beta-actin**	TGCCGCACTGGTTGTTGACAACG	CCATGACACCCTGGTGCCTGG
**Kita**	AGCAACAGCTGTCAGACTCC	GGAGCAGTAAGGGCTGTGTT
